# Supplementary material for: The mutational landscape of Staphylococcus aureus during colonisation
Source: Nat Commun. 2025 Jan 13;16:302. doi: 10.1038/s41467-024-55186-x (PMC11730646; doi:10.1038/s41467-024-55186-x)
Supplement: Supplementary file 1 — Supplementary Information [file 41467_2024_55186_MOESM1_ESM.pdf]

Supplementary Materials

The mutational landscape of *Staphylococcus aureus* during colonisation

Francesc Coll, Beth Blane, Katherine Bellis, Marta Matuszewska, Toska Wonfor, Dorota Jamrozy, Michelle Toleman, Joan A Geoghegan, Julian Parkhill, Ruth C Massey, Sharon J Peacock, Ewan M Harrison

Supplementary Tables

Supplementary Table 1. Collections with multiple sequenced colonising isolates available from the same individual used in this study

Supplementary Table 2. Protein-altering mutations in *nasD* (*nirB*) identified in colonising isolates of the same host

Supplementary Table 3. Putative adaptive mutations in genes encoding for antibiotic targets identified in colonising isolates of the same host

Supplementary Table 4. Reported daptomycin-resistant mutations in *S. aureus*

Supplementary Table 5. Hypothesised daptomycin adaptive mutations in colonising isolates of the same host

Supplementary Table 6. Loss-of-function mutations in *pstS* and *vraA* genes found in a collection of 2,345 MRSA isolates

Supplementary Table 7. Protein-altering mutations in the AgrCA two-component system identified in colonising isolates of the same host

Supplementary Table 8. Collections with multiple sequenced colonising isolates available from the same individual identified after June 2019

Supplementary Figures

Supplementary Figure 1. Selection criteria used to identify collections of multiple colonising isolates sequenced per host

Supplementary Figure 2. Genetic diversity between colonising isolates of the same host

Supplementary Figure 3. Density of mutations attributable to recombination

Supplementary Figure 4. Growth curves of *S. aureus nasD* and *ureG* knock-out mutants under different nitrogen sources

Supplementary Figure 5. Growth curves of *S. aureus nasD* (*nirB)* mutants under different nitrogen sources

Supplementary Figure 6. Protein-altering mutations detected in known antibiotic targets

Supplementary Figure 7. Growth curves of *pstS* and *vraA* mutant and wildtype *S. aureus* clinical isolates under daptomycin exposure

Supplementary Figure 8. Loci enriched for protein-altering mutations in the extended dataset

Supplementary Figure 9. Positions of protein altering mutations on SraP

Supplementary files:

Supplementary Data 1. Isolate accession and metadata

Supplementary Data 2. Hits of mutation enrichment analyses

Supplementary Data 3. Raw growth curves measurements and growth parameters obtained under different nitrogen sources

Supplementary Data 4. Raw growth curves measurements and growth parameters obtained with and without daptomycin

Supplementary Data 5. Mutations detected by Breseq between the pairs of related isolates from the same host tested *in vitro*

Supplementary Table 1. Collections with multiple sequenced colonising isolates available from the same individual used in this study

Study Accession (Publication)	Isolates used (out of available)	Isolates kept after QC	Sources of Isolation	Years of Isolation	Place of Isolation	Setting/cohort	Number of hosts (after QC)	Median isolates per individual
PRJEB3174[1]	727/2,282	702	Multi-site screen	2012 - 2013	East of England, UK	Health care network	284 (274)	2 (IQR 2-3)
PRJNA324190[2]	1,338/1,977	1,107	Nose, perineum, groin, throat and armpit	2011 - 2012	Brighton, UK	Hospital ICU cohort (inc. HCWs)	261 (225)	3 (IQR 2-6)
PRJNA369475[3]	566/1,163	551	Nasal swabs	2009 - 2013	Oxford and Brighton, UK	Hospital cohort	105 (105)	5 (IQR 5-5)
PRJEB9390[4]	172/383	132	Groin, nose, axilla	2014	Singapore	Hospital and nursing homes	78 (62)	2 (IQR 2-2)
PRJEB2862[5]	159/275	139	Nose, perineum and groin	2010 - 2011	Brighton, UK	Hospital ICU cohort	67 (59)	2 (IQR 2-2)
ERP000130[6]	143/172	96	Multi-site screen	2008	Thailand	Hospital ICU cohort	22 (17)	2 (IQR 2-2)
PRJEB20148[7]	90/262	89	Nasal swabs	2015	Scotland, UK	Healthy community children	18 (18)	5 (IQR 5-5)
PRJEB11177[8]	35/448	22	Nose, throat and perineum	2011 - 2012	London, UK	Hospital cohort	17 (11)	2 (IQR 2-2)
PRJEB2655 and PRJEB7654[9]	97/165	89	Multi-site and nasal swabs	2012 - 2014	Galway, Ireland and Cambridge, UK	Nursing homes	16 (16)	3 (IQR 2-5)
PRJEB4141[10]	170/172	133	Nasal swabs	Not found	England	Veterinary hospital staff	4 (4)	52 (IQR 39-55)
Total	3,497/7,299	3,060					872 (791)	2 (IQR 2-4)

Collections of *S. aureus* genomes used in this study where multiple isolate genomes were available from the same host. Only isolate genomes kept after quality control were used for further analyses. Abbreviations: HCWs, heathcare workers.

Supplementary Table 2. Protein-altering mutations in *nasD* (*nirB*) identified in colonising isolates of the same host

Gene (locus tag)	Chr. position	Nucleotide change	Annotation	Amino acid change	Protein domain*	Patient id (isolate id)	Selected for testing**
nasD/*nirB* (SAOUHSC_02684)	2469191	T > C	missense	p.Glu797Gly	-	young2017-P085 (SRR5250431)	not available
	2469499	G > T	missense	p.Phe694Leu	domain E	price2016-2058578 (SRR3729258)	not available
	2469614	G > A	missense	p.Thr656Ile	domain E	coll2027-381 (8525_1#72)	selected
	2469620	T > C	missense	p.Glu654Gly	domain E	young2017-P095 (SRR5250387)	not available
	2469761	T > C	missense	p.Glu607Gly	domain D	price2016-H240 (SRR3728572)	not available
	2469932	T > C	missense	p.His550Arg	-	price2016-3424520 (SRR3728593)	not available
	2469969	C > T	missense	p.Asp538Asn	-	harrison2016-GR11 (13414_7#15)	not available
	2470227	A > T	missense	p.Cys452Ser	domain C	coll2027-194 (9716_3#67)	selected
	2470557	T > C	missense	p.Ile342Val	domain B	price2016-H149 (SRR3728524)	not available
	2470677	G > A	missense	p.His302Tyr	-	price2016-3414160 (SRR3730410)	not available
	2470845	C > G	missense	p.Glu246Gln	domain A	coll2027-547 (14324_8#22)	selected
	2471340	G > A	missense	p.Pro81Ser	domain A	price2016-H127 (SRR3728586)	not available
	2471465	G > A	missense	p.Pro39Leu	domain A	price2016-H179 (SRR3729445)	not available
	2471562	C > A	missense	p.Val7Leu	domain A	chow2017-CD140187 (17059_1#16)	not available

*Domain referred here: domain A, FAD/NAD(P)-binding domain; domain B, NADH-rubredoxin oxidoreductase domain; domain C, BFD-like [2Fe-2S]-binding domains; domain D, Nitrite/Sulfite reductase ferredoxin-like domain; domain E, Nitrite/sulphite reductase 4Fe-4S domain. **Only isolates from internal collections (i.e. coll2017) were available for testing, see Supplementary Data 5 for all mutations identified between colonising isolates of the same host. Mutations found in the strains of a maximum of 791 individuals (see Supplementary Table 1).

Supplementary Table 3. Putative adaptive mutations in genes encoding for antibiotic targets identified in colonising isolates of the same host

Antibiotic	Gene	Chr. position	Nt. change	Amino acid change	Patient id (isolate id)	Known role*	Phenotypic resistance (MIC)**	Wildtype isolate id	Wildtype isolate MIC **
Fusidic acid	*fusA*	531299	G > A	p.Val90Ile	coll2017-1295 (14623_7#61)	Yes	R (2 μg/mL)	14412_8#14	S (<=0.5 μg/mL)
		531300	T > C	p.Val90Ala	young2017-P040 (SRR5249845)	Yes	Isolate NA	-	-
		531392	C > T	p.Arg121Cys	coll2017-608 (14412_8#87)	No	S (<=0.5 μg/mL)	14355_1#58	S (<=0.5 μg/mL)
		531719	C > T	p.Leu230Phe	coll2017-534 (14672_2#27)	No	S (<=0.5 μg/mL)	14200_6#68	S (<=0.5 μg/mL)
		532242	C > T	p.Pro404Leu	coll2017-254 (8447_7#92)	Yes	R (16 μg/mL)	14200_8#72	S (<=0.5 μg/mL)
		532401	A > G	p.His457Arg	coll2017-1162 (14208_8#9)	Codon	R (>=32 μg/mL)	14623_3#77	R (16 μg/mL)
		532401	A > G	p.His457Arg	coll2017-527 (14208_8#16)	Codon	R (>=32 μg/mL)	14200_6#27	R (16 μg/mL)
		532550	G > A	p.Gly507Ser	coll2017-104 (14448_1#44)	No	S (<=0.5 μg/mL)	14200_8#74	S (<=0.5 μg/mL)
		532770	C > T	p.Ala580Val	coll2017-103 (14355_1#61)	No	S (<=0.5 μg/mL)	8524_3#66	S (<=0.5 μg/mL)
		533048	G > A	p.Asp673Asn	young2017-P094 (SRR5249970)	No	Isolate NA	-	-
Mupirocin	*ileS*	1107196	A > G	p.Lys23Glu	price2016-H277 (SRR3728914)	No	Isolate NA	-	-
		1108541	DEL.[2]	p.Ile473fs	coll2017-121 (14324_8#70)	No	R (1,024 μg/mL)§	8490_3#36	S (0.19 μg/mL)§
		1108891	G > T	p.Val588Phe	coll2017-125 (14623_6#41)	Yes	R (>=512 μg/mL)	14200_8#87	S (<=2 μg/mL)
		1108891	G > T	p.Val588Phe	coll2017-291 (14208_8#53)	Yes	I (12 μg/mL) §	8524_5#51	S (0.19 μg/mL)§
		1108900	G > A	p.Gly591Ser	coll2017-520 (14355_1#12)	No	S (0.5 μg/mL) §	14200_6#18	S (0.19 μg/mL)§
		1108907	G > C	p.Gly593Ala	price2013-X035 (SRR2053963)	Codon	Isolate NA	-	-

		1109020	G > T	p.Val631Phe	coll2017-177 (14623_8#7)	Yes	I (3 µg/mL)§	14324_8#86	S (0.19 µg/mL)§
		1109020	G > T	p.Val631Phe	coll2017-75 (14623_8#86)	Yes	I (12 µg/mL)§	-	-
Trimethoprim	*dfrA*	1370041	T > C	p.His150Arg	coll2017-789 (14672_2#49)	Yes	S (<=0.5 µg/mL, 27 mm)	14200_7#34	S (<=0.5 µg/mL, 28 mm)
		1370098	T > C	p.Asp131Gly	price2016-H213 (SRR3730594)	No	Isolate NA	-	-
		1370180	C > A	p.Asp104Tyr	chow2017-CD140174 (17059_1#8)	No	Isolate NA	-	-
		1370194	A > T	p.Phe99Tyr	price2016-H264 (SRR3730705)	Yes	Isolate NA	-	-
		1370194	A > T	p.Phe99Tyr	coll2017-237 (14623_6#13)	Yes	R (>=16 µg/mL)	8447_7#65	S (<=0.5 µg/mL)
Beta-lactams	*pbp2*	1421571	C > A	p.Ser7Tyr	price2016-2107188 (SRR3728791)[3]	-	Isolate NA	-	-
		1421757	T > C	p.Leu69Ser	chow2017-CD140955 (16870_4#6)[4]	-	Isolate NA	-	-
		1421900	C > T	p.Arg117Cys	young2017-P069 (SRR5251343)[3]	-	Isolate NA	-	-
		1421900	C > A	p.Arg117Ser	coll2017-292 (8524_5#53)[4]	-	CFX R (>4 µg/mL)	14623_2#62	CFX R (>4 µg/mL)
		1421933	C > A	p.Arg128Ser	young2017-P069 (SRR5250494)[3]	-	Isolate NA	-	-
		1421972	T > G	p.Phe141Val	coll2017-547 (14324_8#22)[4]	-	CFX S (<=4 µg/mL)	14200_8#21	CFX R (>4 µg/mL)
		1421976	G > A	p.Gly142Asp	young2017-P080 (SRR5250797)[3]	-	Isolate NA	-	-
		1421994	C > T	p.Thr148Ile	young2017-P079 (SRR5250247)[3]	-	Isolate NA	-	-
		1422237	G > C	p.Gly229Ala	young2017-P006 (SRR5249848)[3]	-	Isolate NA	-	-
		1422256	C > A	p.Asn235Lys	coll2017-150 (8490_3#60)[4]	-	CFX S (<=4 µg/mL)	14448_3#45	CFX R (>4 µg/mL)

* Known antibiotic-resistant mutations were extracted from Kumar *et al.* 2020[11].

** Isolates from ‘coll2017-’ patients (internal collection) had available AST VITEK data and were available for further antibiotic susceptibility testing, see Supplementary Data 5 for all mutations identified between colonising isolates of the same host. A few

isolates were re-tested with disc diffusion, for which zone diameters are indicated in millimetres. §MIC confirmed using E-test. Isolate NA: isolate not available for re-testing. Mutations found in the strains of a maximum of 791 individuals (see Supplementary Table 1). [2] Deletion: GCGAAATTATCATGA > G. [3] MSSA strain (lacking both *mecA* and *mecC*). [4] MRSA strain with *mecA*. Abbreviations: MIC, Minimum Inhibitory Concentration; CFX, cefoxitin; AST, antibiotic susceptibility testing.

Supplementary Table 4. Reported daptomycin-resistant mutations in *S. aureus*

Gene	Mutations reported in the literature	Locus tag (hit position)	Mutations in within-host dataset
mprF	T345A[12–17], P314L[13,18,19], T345I[13,14,18,20–22], T345X, V351E[23], S295L[19,22,24,25], L338S[26], S337L[14,18,19,22,26–28], L776S[14], A475P[14], L459_H466 del[14], L291I[14], W424R[14], L341S[14], P314L[19], S295A[27], I348del[27], R50L[18], R301L[18], L425F[18], P314L[29], L826F[14,19,22,29–31], L826I[19], S829L[32], H376Y&W424C[19], A302V[19], M347R[19], 41insN[19], M347L[33], L770F[33], I420N[22], G61V[22]<br>Increased expression[16,33]	SAOUHSC_01359<br>19th hit	p.Arg50Cys, p.Gly61Val, p.Gly61Val, p.Ser156Tyr, p.Met323Ile, p.Glu593Asp, p.Ala715Glu, p.Ala727Thr, p.Ser829Leu, p.Ser829Leu
yycF	K151N[19]	SAOUHSC_00020<br>Not detected	Not extracted
yycG	S221P[13], R263C[13,15,17], I568V[26], I185T[26], Q51K[26], G199E[19], 369delQ[19], G223D[34], M426I[31]<br>Increased expression[35]	SAOUHSC_00021<br>354th	Not extracted
rpoB	I953S[13], A1086V[13,15,17] L770F[33],Q468K[36], A477D[25,36], H481N[36], H481Y[16,23], H481R[16], S464P[36], A621E[16,37] R484C&N641K[34], D471Y&A473S&A477S&E478D[34]	SAOUHSC_00524<br>1421st	Not extracted
rpoC	F632S[13], Q961K[13,15,17], N735K[19]	SAOUHSC_00525<br>1068th	Not extracted

vraS	Increased expression[25,26,30,38,39]<br>T331I[32], G45V[38], E276K[40], L114S&D242G[41]	SAOUHSC_02099<br>2035th	Not extracted
vraR	Increased expression[25,30,33,39]	SAOUHSC_02098<br>2034th	Not extracted
vraT	A151T[42]	SAOUHSC_02100<br>1007th	Not extracted
tagH	A39T[42]	SAOUHSC_02009<br>1633rd	Not extracted
cls2	R320L[19], R320S[19], F85X[19], L77F[19], A56G&T58N[19], A23V[22,43], T33N[43], L52F[22,43], F60[22,43], E38G[21], L190F[21]<br>Increased expression[44]	SAOUHSC_02323<br>915th	Not extracted
pgsA	A64V[22,45], S177F[22,45], K65R[22], V59N[22,45], V59D[45], G61S[45], K75N[45], K135E[31,45], D187E[45]	SAOUHSC_01260<br>327th	Not extracted
ddl	No mutations found	SAOUHSC_02318<br>1252nd	Not extracted
dltA	A426E[19]<br>Increased expression[16,44,46]	SAOUHSC_00869<br>49th	p.Ser37Ala<br>p.Leu219Ile<br>p.Asp306Tyr<br>p.Thr310Ile<br>p.Pro467Ser<br>p.Val482Ala
clpX	A348V[21], nt168_del[25]	SAOUHSC_01778<br>177th	Not extracted

Mutations reported to be associated be daptomycin non-susceptibility in the literature. The hit position of candidate genes in the CDS mutation enrichment analysis is presented in the third column, along with the mutations found in this dataset in the fourth column.

Supplementary Table 5. Hypothesised daptomycin adaptive mutations in colonising isolates of the same host

Gene (locus tag)	Chr. position	Nucleotide change	Annotation	Amino acid change	Protein domain*	Patient id (isolate id)	Selected for testing	Dap. MIC	Daptomycin MIC of wildtype isolate**
pstS (SAOUHSC_01389)	1331476	C > T	missense	p.Ala291Thr	-	price2016-2754372 (SRR3731418)	NA	NA	NA
	1331536	A > G	missense	p.Phe271Leu	domain A	price2016-H123 (SRR3731460)	NA	NA	NA
	1331730	C > T	missense	p.Gly206Glu	domain A	coll2027-359 (8525_1#49)	Yes	0.38	0.5 (14208_8#2)
	1331764	C > T	missense	p.Ala195Thr	domain A	young2017-P048 (SRR5250629)	NA	NA	NA
	1331852	TGGTG > T	frameshift	p.Ser164fs	domain A	coll2027-64 (14200_8#1)	Yes	0.38	0.38 (8524_3#24)
	1331853	G > T	missense	p.Pro165Gln	domain A	price2016-H187 (SRR3729045)	NA	NA	NA
	1332186	T > C	missense	p.Glu54Gly	domain A	young2017-P046 (SRR5250023)	NA	NA	NA
vraA (SAOUHSC_00557)	566249	C > T	missense	p.Pro60Ser	domain A	coll2027-97 (8524_3#62)	Yes	0.19	0.5 (14448_1#41)
	566255	C > G	missense	p.Gln62Glu	domain A	chow2017-CD140187 (17059_1#17)	NA	NA	NA
	566441	C > A	missense	p.His124Asn	domain A	price2016-H122 (SRR3729370)	NA	NA	NA
	566794	A > AT	frameshift	p.Ser244fs	domain A	young2017-P009 (SRR5250318)	NA	NA	NA
	567014	A > G	missense	p.Ile315Val	domain A	harkins2018-SS_157 (ERR1904049)	NA	NA	NA
	567321	T > A	stop gained	p.Leu417*	domain B	coll2027-381 (14208_8#20)	Yes	0.75	0.38 (8525_1#72)
	567321	TA > T	frameshift	p.Lys419fs	domain B	paterson2015-Staff_D (10770_3#44)	Yes	0.75	0.38 (10900_1#28)
	567390	C > A	missense	p.Ala440Glu	domain B	chow2017-CD140764 (16870_3#11)	NA	NA	NA

*pstS contains a single PBP superfamily domain (http://pfam.xfam.org/protein/Q2FYP6), here labelled as domain A. vraA is made up of two domains (http://pfam.xfam.org/protein/Q2G0K3): an AMP-binding enzyme domain (here labelled as domain A) and an AMP-binding enzyme C-terminal domain (domain B). ** isolates from the same host without mutation. Mutations found in the strains of a maximum of 791 individuals (see Supplementary Table 1).

Supplementary Table 6. Loss-of-function mutations in *pstS* and *vraA* genes found in a collection of 2,345 MRSA isolates

Gene (locus tag)	Chr. position	Nuc. change	Annotation	Amino acid change[1]	Protein domain[2]	isolate id	Dap. MIC[3]	Related isolate	Genetic distance (SNPs)	Related isolate Dap. MIC[3]	Known dap. mutations
pstS (SAOUHSC_01389)	1387079	A > AT	frameshift	p.Ter328fs	-	14355_1#80	ND	14623_5#49	73	ND	-
	1387079	A > AT	frameshift	p.Ter328fs	-	14623_7#5	ND	14623_5#49	83	ND	-
	1387339	GT > G	frameshift	**p.Thr241fs**	domain A	14324_8#29	0.5	14448_2#11	16	0.5	-
	1387339	G > GT	frameshift	**p.Thr241fs**	domain A	14623_5#47	ND	14355_2#16	86	ND	-
	1387339	GT > G	frameshift	**p.Thr241fs**	domain A	14672_2#74	ND	14623_3#79	53	ND	-
	1387339	GT > G	frameshift	**p.Thr241fs**	domain A	8447_7#72	ND	14623_3#79	52	ND	-
	1387412	G > A	stop gained	p.Gln217*	domain A	14412_8#80	0.5	14355_1#89	16	0.5	-
	1387491	A > AT	frameshift	**p.Asn190fs**	domain A	14355_1#57	ND	14623_2#41	72	ND	-
	1387491	A > AT	frameshift	**p.Asn190fs**	domain A	14355_2#77	ND	14623_2#41	74	ND	-
	1387491	AT > A	frameshift	**p.Asn190fs**	domain A	14448_3#24	0.25	14200_6#37	42	0.5	-
	1387491	A > AT	frameshift	**p.Asn190fs**	domain A	14555_7#38	ND	14623_2#41	74	ND	-
	1387491	AT > A	frameshift	**p.Asn190fs**	domain A	14623_7#4	0.38	14200_6#37	41	0.5	-
	1387566	TGGTG > T	frameshift	p.Ser164fs	domain A	14200_8#1	ND	14623_8#85	23	ND	-
	1387582	A > AT	frameshift	p.Ile160fs	domain A	14623_4#78	ND	14323_1#84	66	ND	-
	1387954	TC > T	frameshift	p.Glu36fs	domain A	14623_3#1	ND	8524_5#62	45	ND	-

vraA (SAOUHSC_ 00557)	601292	C > T	stop gained	p.Gln13*	domain A	14208_8#78	1.5	14200_6#58	76	0.064	*mprF* p.Thr345Ala
	601292	C > T	stop gained	p.Gln13*	domain A	14623_8#74	2	14200_6#58	71	0.064	*mprF* p.Thr345Ala
	601292	C > T	stop gained	p.Gln13*	domain A	8447_7#54	1.5	14200_6#58	60	0.064	*mprF* p.Thr345Ala
	601616	C > T	stop gained	p.Gln121*	domain A	14200_8#40	0.25	8525_1#80	84	0.38	-
	601629	AT > A	frameshift	p.Asn127fs	domain A	14412_8#43	ND	8490_3#77	546	ND	-
	601629	AT > A	frameshift	p.Asn127fs	domain A	14448_4#53	ND	8490_3#77	575	ND	-
	601809	T > A	stop gained	p.Leu185*	domain A	14623_3#35	0.38	14200_8#1	69	0.38	-
	601809	T > A	stop gained	p.Leu185*	domain A	14623_3#89	0.25	14200_8#1	69	0.38	-
	601844	C > T	stop gained	p.Gln197*	domain A	14623_6#73	ND	14355_2#73	202	ND	-
	602186	G > T	stop gained	p.Gly311*	domain A	14412_8#68	0.5	8490_3#55	65	0.38	-
	602186	G > T	stop gained	p.Gly311*	domain A	14623_6#28	0.38	8490_3#55	65	0.38	-
	602186	G > T	stop gained	p.Gly311*	domain A	14623_7#77	ND	8490_3#55	68	ND	-
	602286	TAA > T	frameshift	p.Lys345fs	domain A	14355_1#1	ND	14355_2#9	532	ND	-
	602286	TAA > T	frameshift	p.Lys345fs	domain A	14355_1#3	ND	14355_2#9	500	ND	-
	602286	TAA > T	frameshift	p.Lys345fs	domain A	14623_4#87	ND	14355_2#9	500	ND	-
	602286	TAA > T	frameshift	p.Lys345fs	domain A	8447_7#44	ND	14355_2#9	500	ND	-
	602286	TAA > T	frameshift	p.Lys345fs	domain A	8447_7#45	ND	14355_2#9	500	ND	-
	602286	TAA > T	frameshift	p.Lys345fs	domain A	8490_3#61	ND	14355_2#9	500	ND	-
	602505	T > A	stop gained	**p.Leu417***	domain B	14208_8#20	ND	8525_1#72	25	ND	-
	602505	T > A	stop gained	**p.Leu417***	domain B	14623_7#13	ND	none found	NA	ND	-

[1]Mutations in bold were found to be homoplasic. [2]See information about vraA and pstS protein domains in the footnote of Supplementary Table 5. [3]Daptomycin MICs were determined using E-test. For each isolate, the genetically closest isolate was selected. Not all isolates

were selected for daptomycin susceptibility testing. Of all detected loss-of-function mutations, isolates were selected if the carried a nonsense (stop gained) mutation or homoplasic frameshift mutation, and had a genetically related isolate below 100 SNPs. If multiple isolates carrying the same mutation formed a monophyletic clade, then no more than two isolates with such mutation were selected for testing. *vraA* LOF mutations did not lead to a measurable increase in daptomycin MIC with the exception of isolates carrying also a well-known daptomycin-resistance conferring mutation (*mprF* p.Thr345Ala). See Supplementary Table 7 for a comprehensive list of daptomycin-resistance conferring mutations. Mutations found in a collection of 2,345 MRSA isolates from 1,465 individuals (see [1]).

Supplementary Table 7. Protein-altering mutations in the AgrCA two-component system identified in colonising isolates of the same host

Gene (locus tag)	Chr. position	Nucleotide change	Annotation	Amino acid change	Protein domain	Patient id (isolate id)	Selected for testing (criterium)*
agrC (SAOUHSC_02264)	2094797	TTTA > T	DID	p.Ile52del	TMD 2	chow2017-CD140408 (17059_1#56)	not available
	2094952	C > CT	frameshift	p.Val103fs	TMD 4	tong2015-T197 (4950_3#3)	not available
	2094974	AT > A	frameshift	p.Ser109fs	TMD 4	coll2017-675 (14412_8#81)	selected (TMD, frameshift)
	2095085	A > G	missense	p.Thr145Ala	TMD 5	coll2017-308 (14448_2#30)	not selected
	2095306	G > A	missense	p.Met218Ile	DHp (HK)	coll2017-191 (14355_2#69)	not selected
	2095340	AC > A	frameshift	p.Thr230fs	DHp (HK)	coll2017-624 (14355_1#90)	not selected
	2095399	TA > T	frameshift	p.Asn251fs	DHp (HK)	coll2017-257 (8524_5#5)	not selected
	2095409	G > A	missense	p.Val253Ile	DHp (HK)	coll2017-416 (14324_8#66)	selected (DHp, missense)
	2095435	G > A	missense	p.Met261Ile	DHp (HK)	coll2017-79 (14623_2#2)	not selected
	2095501	GA > G	frameshift	p.Ile285fs	DHp (HK)	coll2017-273 (8524_5#30)	not selected
	2095517	C > T	stop gained	p.Gln289*	DHp (HK)	coll2017-571 (14623_3#73)	not selected
	2095556	G > T	stop gained	p.Glu302*	DHp (HK)	coll2017-764 (14324_8#39 & 14355_2#91)**	selected (DHp, stop codon)
	2095556	G > T	stop gained	p.Glu302*	DHp (HK)	coll2017-571 (14623_3#73)	not selected
	2095592	AGTC > A	DID	p.Arg315del	CA (HK)	coll2017-132 (14448_1#76)	selected (CA, inframe deletion)
	2095605	G > T	missense	p.Gly318Val	CA (HK)	coll2017-1232 (14623_4#15)	not selected

	2095668	C > T	missense	p.Ala339Val	CA (HK)	auguet2016-P_0130 (ERR1040796)	not available
	2095711	T > TA	frameshift	p.Cys355fs	CA (HK)	coll2017-1263 (14623_7#55)	selected (CA, frameshift)
	2095724	GA > G	frameshift	p.Asp358fs	CA (HK)	chow2017-CD141173 (16870_4#49)	not available
	2095785	G > A	missense	p.Gly378Asp	CA (HK)	coll2017-547 (14200_8#21)	selected (CA, missense)
	2095808	G > T	stop gained	p.Glu386*	CA (HK)	price2013-X077 (SRR2054058)	not available
	2095836	T > A	stop gained	p.Leu395*	CA (HK)	price2016-H211 (SRR3730915)	not available
agrA (SAOUHSC_02265)	2096082	A > G	missense	p.Tyr26Cys	RR	coll2017-450 (14200_6#83)	not selected
	2096118	G > A	missense	p.Gly38Asp	RR	coll2017-719 (14355_2#54)	selected (RR, missense)
	2096130	G > A	missense	p.Gly42Asp	RR	price2016-H213 (SRR3728573, SRR3728579, SRR3729029 & SRR3730594)**	not available
	2096292	CT > C	frameshift	p.Ala97fs	ID	coll2017-364 (14412_8#75)	selected (ID, frameshift)
	2096358	G > A	missense	p.Gly118Asp	DNA BD	price2016-3449573 (SRR3729415)	not available
	2096358	G > A	missense	p.Gly118Asp	DNA BD	coll2017-1295 (14412_8#14)	selected (DNA BD, missense)
	2096390	A > G	missense	p.Ile129Val	DNA BD	coll2017-95 (8524_3#54)	not selected
	2096395	GT > G	frameshift	p.Phe132fs	DNA BD	coll2017-261 (14355_1#24, 14555_7#35 & 14672_2#53)**	not selected
	2096395	G > GT	frameshift	p.Glu133fs	DNA BD	coll2017-254 (14200_8#72)	selected (DNA BD, frameshift)
	2096395	G > GT	frameshift	p.Glu133fs	DNA BD	coll2017-503 (14623_8#17)	not selected
	2096395	G > GT	frameshift	p.Glu133fs	DNA BD	coll2017-377 (14623_5#51)	not selected
	2096418	C > A	missense	p.Ser138Tyr	DNA BD	price2016-H153 (SRR3728921)	not available

	2096447	C > T	missense	p.Arg148Cys	DNA BD	price2013-X168 (SRR2054034)	not available
	2096462	T > C	missense	p.Tyr153His	DNA BD	price2016-H122 (SRR3730764, SRR3729005 & SRR3731680)**	not available
	2096499	G > A	missense	p.Arg165His	DNA BD	price2013-X054 (SRR2054010)	not available
	2096562	A > T	missense	p.Lys186Ile	DNA BD	coll2017-190 (8447_7#11 & 14672_2#5)**	selected (DNA BD, missense)
	2096567	C > T	stop gained	p.Arg188*	DNA BD	young2017-P042 (SRR5251255)	not available
	2096582	A > T	stop gained	p.Lys193*	DNA BD	price2016-H236 (SRR3728784)	not available
	2096590	41nt > A	frameshift	p.Glu196fs	DNA BD	harrison2016-GR13 (13414_7#6)	not available
hld-argB intergenic region	2093655	C > T (-178)	-	-	-	chow2017-CD142136 (17150_1#50)	not available
	2093784	C > T (-49)	-	-	AgrA BS	coll2017-257 (14623_8#22)	selected (intergenic)
	2093801	C > T (-32)	-	-	AgrA BS	price2016-2754372 (SRR3731418)	not available
	2093812	G > A (-21)	-	-	AgrA BS	price2016-2633534 (SRR3731564)	not available
	2093812	G > A (-21)	-	-	AgrA BS	harkins2018-SS_094 (ERR1904013)	not available

*Only isolates from internal collections (i.e. coll2017) were available for testing, see Supplementary Data 5 for all mutations identified between colonising isolates of the same host. **For a total of 5 different individuals, the same Agr mutation was detected in multiple isolates, each one isolated in independent swabs taken at different time points. See isolate collection dates in Supplementary Data 1. One isolate representative of each type of mutation (missense, frameshift and stop gained) at domain were selected for further testing. Mutations found in the strains of a maximum of 791 individuals (see Supplementary Table 1). Abbreviations: AgrA BS, AgrA Binding Site (AgrA tandem repeats); CA, Catalytic and ATP-binding subdomain of the Histidine Kinase domain; Dimerization and Histidine phosphotransfer subdomain of the Histidine Kinase domain; DID, disruptive inframe deletion; DNA BD, DNA Binding Domain; ID, Inter Domain (region between domains); RR, Response Regulator domain; TMD, transmembrane domain.

Supplementary Table 8. Collections with multiple sequenced colonising isolates available from the same individual identified after June 2019

Study Accession (Publication)	Isolates used (out of available)	Isolates kept after QC	Sources of Isolation	Years of Isolation	Place of Isolation	Setting/cohort	Number of hosts	Median isolates per individual
PRJEB4140, PRJEB2076 & PRJEB2489[47]	1865/1935	457	Nasal, throat and axilla swab	2008	Ubon Ratchathani, Thailand	Hospital ICU cohort	34	14.5 (2-19.5)
PRJEB9390[48]	717/1478	391	Axilla, groin and nasal swab	2014 - 2016	Singapore	Hospital and nursing homes	186	2 (2-2)
PRJEB28206[49]	318/784	282	Nasal swabs	2018 - 2019	Cambridge, UK	Healthy school children	141	2 (2-2)
PRJDB5246[50]	124/242	60	Cheek skin	2010 - 2014	Chiba, Japan	Healthy neonates	30	2 (2-2)
PRJEB33854[51]	35/37	23	Nasal swab	2016	Westphalia, Germany	Healthy pig farm workers	6	3 (3-4.5)
PRJEB40888[52]	103/165	60	Axilla, groin and nasal swab	2012 - 2014	Australia	Dialysis clinics cohort	18	2.5 (2-3.75)
PRJEB43023[53]	154/155	76	Nasal swabs	2012 - 2014	Denmark	Healthy pig truck drivers	14	5 (2-7.75)
PRJNA590514[54]	254/313	216	Groin and nasal swab	2017 - 2018	Maryland, USA	Nursing home	74	3 (2-3.75)
PRJNA587530[55]	199/244	151	Nasal, throat, groin, and perianal swabs	2015	Georgia, USA	Infantry soldiers	37	3 (2-5)
PRJNA595570[56]	96/127	40	Nasal, hands, throat, groin, axilla, and perianal swabs	2015 - 2016	Chicago, Illinois, USA	Hospital ICU cohort (inc. HCWs)	18	2 (2-2)
PRJNA530184[57]	191/267	115	Nasal, throat and groin swabs	2016 - 2017	Chicago, Illinois, USA	Prison	47	2 (2-3)
PRJNA638400[58]	50/220	38	Nasal, throat and groin swabs	2007 - 2017	Midwest, USA	Health-care network	16	2 (2-3)
PRJNA715375, PRJNA715649 & PRJNA816913[59]	382/1531	112	Nasal swab	2017 - 2018	Mexico City, Mexico	Children dermatology clinic	19	5 (3-6.5)
PRJNA685142[60]	97/140	68	Pooled four-site surveillance swab	2017 - 2018	New York City, USA	Hospital NICU cohort	26	2 (2-3)

PRJNA918392[61]	3675/3818	2001	Nasal, throat and groin swabs	Not specified	Pennsylvania, USA	Unknown	136	11 (7.75-20.25)
Total	8260/11456	4090					802	2 (2-4)

See footnote of Supplementary Table 1 for legend information.

Supplementary Figure 1 Selection criteria used to identify collections of multiple colonising isolates sequenced per host

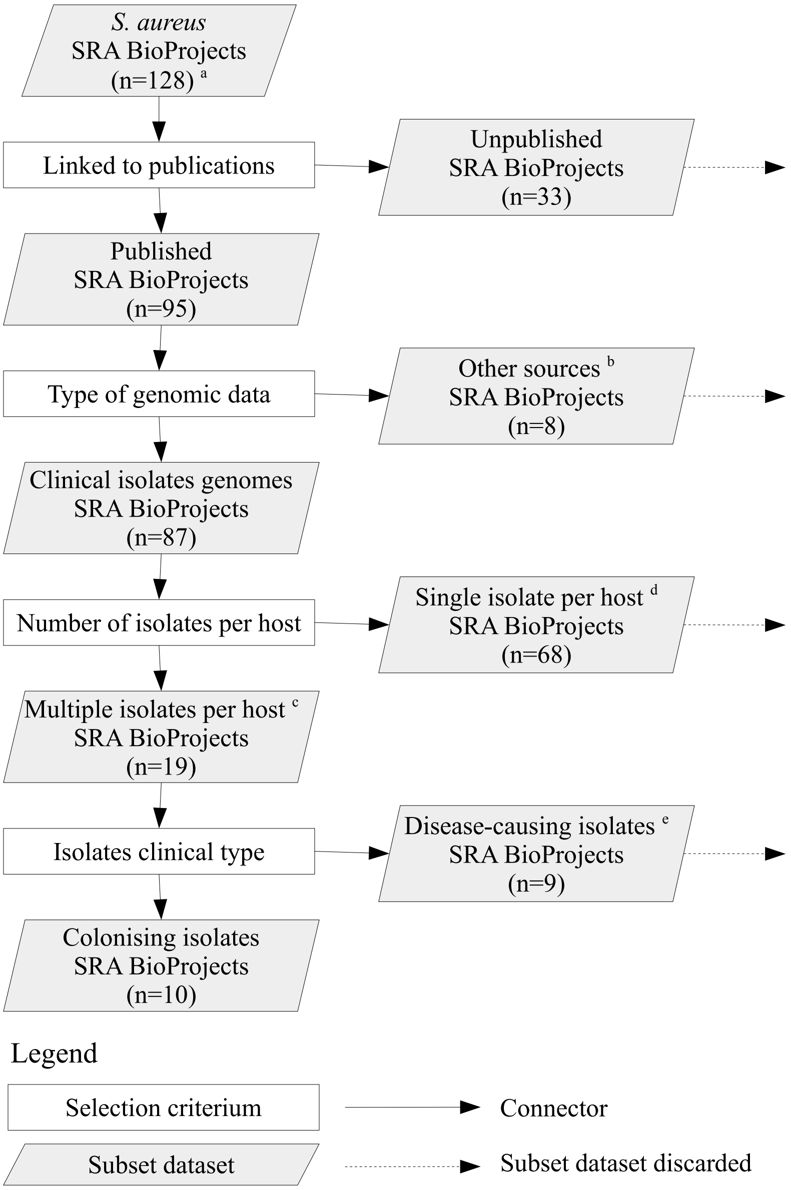

A total of 10 collections (SRA BioProjects) with multiple carriage isolates sequenced from the same host were identified after querying systematically for all *S. aureus* Short Read Archive (SRA) genomic data published to date (searched done by June 2019). [a] BioProjects with less than 70 *S. aureus* isolate genomes were discarded. [b] BioProjects including genomic data other than *S. aureus* clinical isolate genomes; that is, animal strains, mutagenesis experiments, RNAseq or microbiomes were discarded. [c] Host identifiers also had to be available in metadata to link isolates to their corresponding host. [d] BioProjects with multiple isolates per host but without host identifiers could not be included. [e] Datasets with multiple disease-causing isolates per host, or datasets with both colonising and disease-causing isolates per host but with only a single colonising isolate per host, were discarded.

Supplementary Figure 2 Genetic diversity between colonising isolates of the same host

A

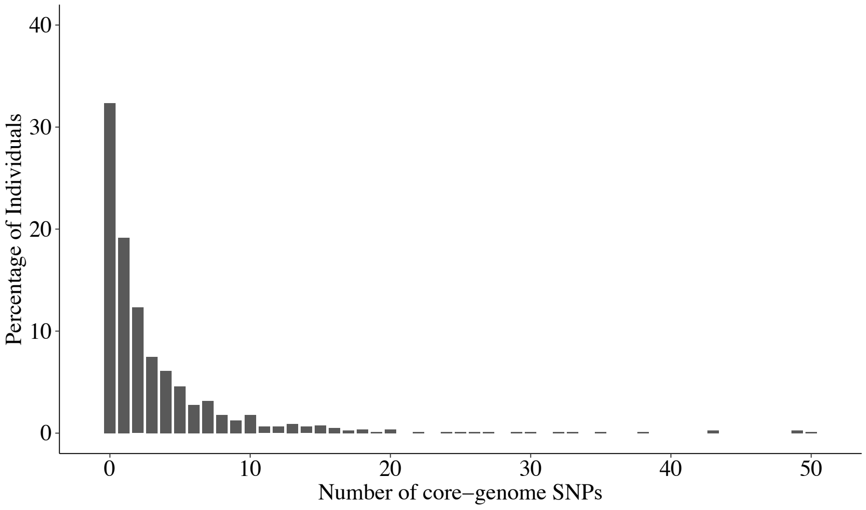

B

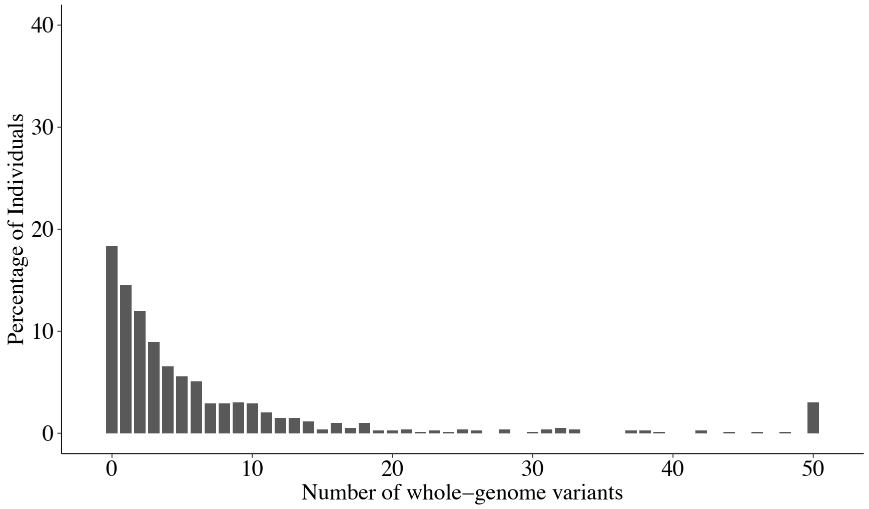

A. Genetic diversity measured as the maximum number of core-genome SNPs observed between isolates of the same individual. B. Genetic diversity measured as the total number of different genetic variants (SNPs and indels) in the whole genome observed across all isolates from the same individual. In the y-axis, percentage of individuals (out of total of 791) having the number of genetic variants specified in the x-axis.

Supplementary Figure 3. Density of mutations attributable to recombination

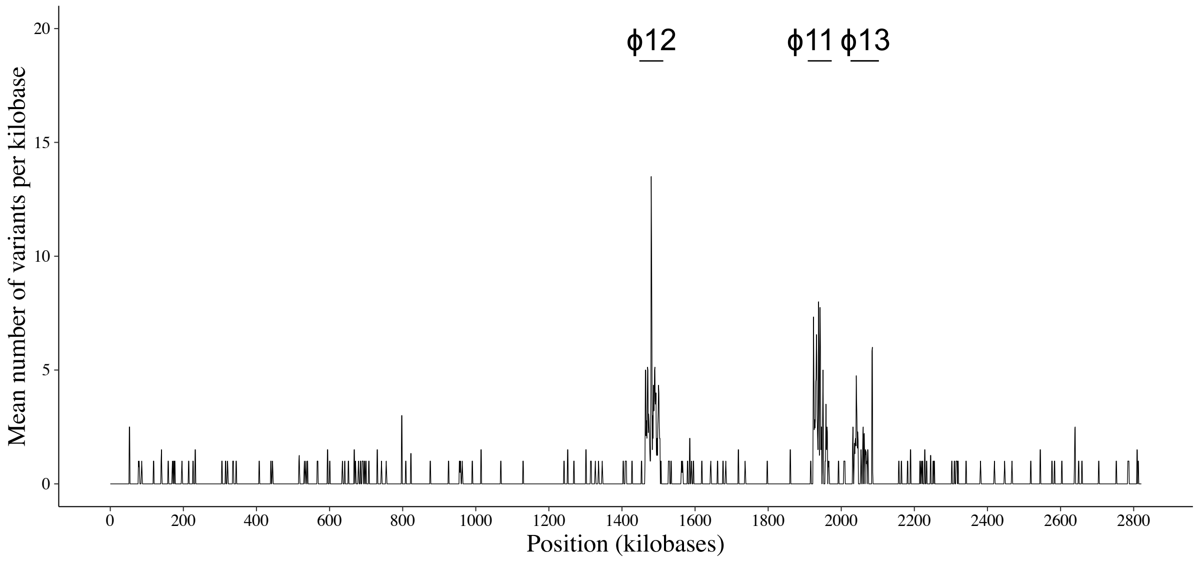

A sliding window of 2 kb was used to calculate the density of mutations attributable to recombination. The mean number of variants per kilobase is plotted along the NCTC8325 reference genome. Most recombination is concentrated in three regions of the genome annotated as prophages phi 11 (AF424781.1), phi 12 (AF424782.1) and phi 13 (AF424783.1).

Supplementary Figure 4 Growth curves of S. aureus *nasD* and *ureG* knock-out mutants under different nitrogen sources

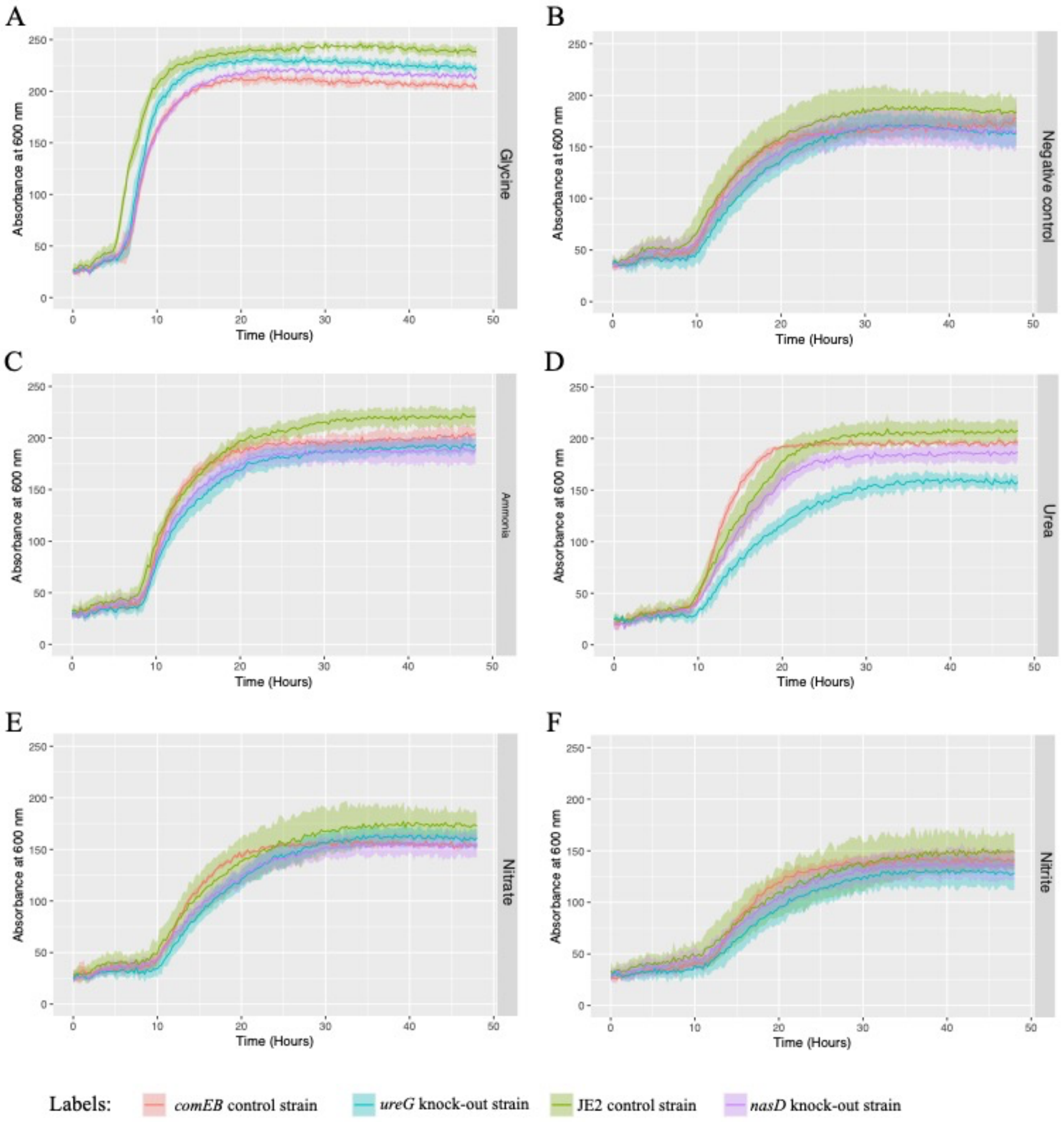

Growth curves of *S. aureus nasD*/*nirB*, *ureG* and *comEB* knock-out mutants; and JE control strain under the following nitrogen sources: glycine (panel A), negative control well (B), ammonia (C), urea (D), nitrate (E) and nitrite (F). Coloured lines represent mean OD600 calculated across three replicates, and shaded coloured regions the standard deviation.

Supplementary Figure 5. Growth curves of *S. aureus nasD*(*nirB*) mutants under different nitrogen sources

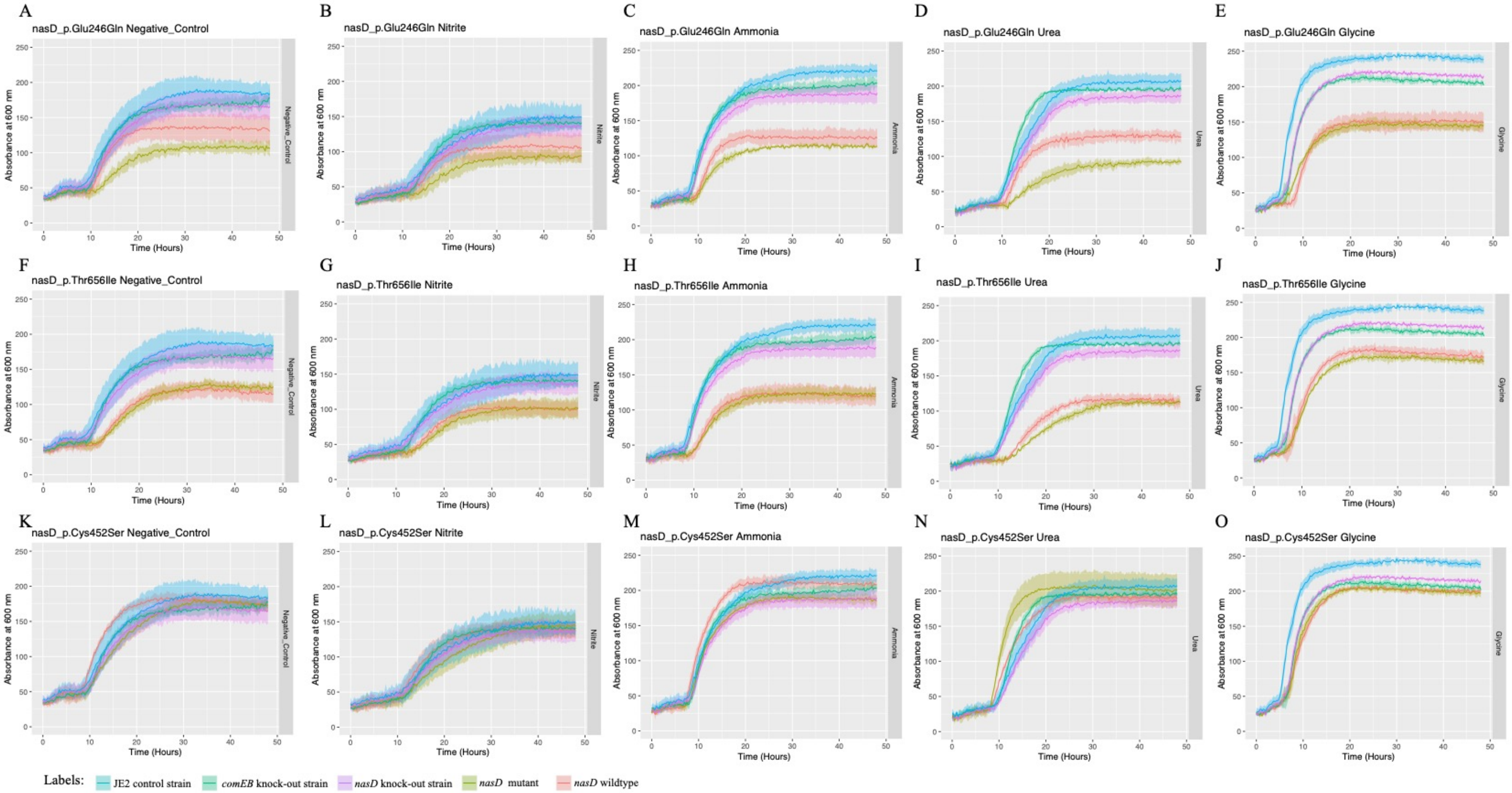

Growth curves of *S. aureus nasD/nirB* mutants, wildtypes (i.e. quasi-isogenic isolate lacking the *nasD/nirB* mutation from the same host), *nasD/nirB* knock-out, *comEB* knock-out (control) and JE (control) strain under the following nitrogen sources: negative control well, nitrite, ammonia, urea and glycine. Coloured lines represent mean OD600 calculated across three replicates, and shaded coloured regions the standard deviation.

Supplementary Figure 6. Protein-altering mutations detected in known antibiotic targets.

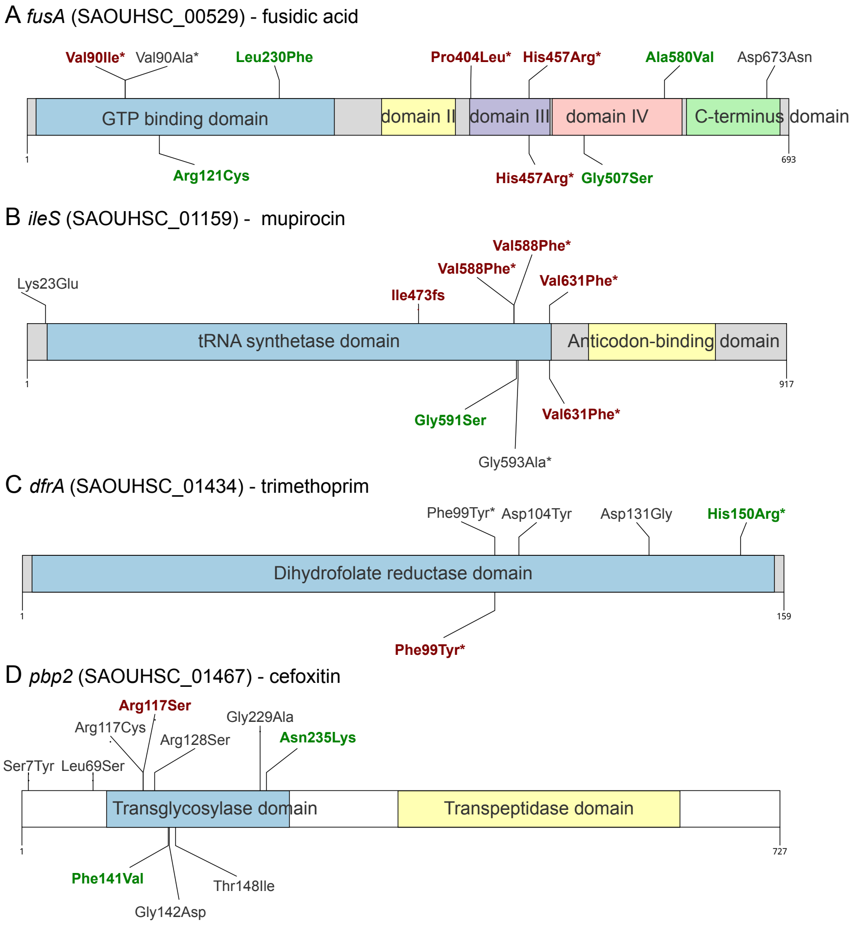

The proteins and protein domains of genes encoding antibiotics targets are shown. Mutations linked to a decreased susceptibility (increase in MIC) to their cognate antibiotic are coloured in red, while mutations in susceptible isolates are shown in green. Non-coloured mutations were found in external isolates that could not be tested. Asterisks indicate mutations reported to confer antibiotic resistance. (A) Elongation factor G, the target of fusidic acid, encoded by *fusA*. (B) Isoleucyl-tRNA synthetase, the target of mupirocin, encoded by *ileS*. (C) Dihydrofolate reductase, the target of trimethoprim, encoded by *dfrA*. (D) Penicillin-binding protein 2, target of beta-lactams, encoded by *pbp2*.

Supplementary Figure 7. Growth curves of *pstS* and *vraA* mutant and wildtype *S. aureus* clinical isolates under daptomycin exposure.

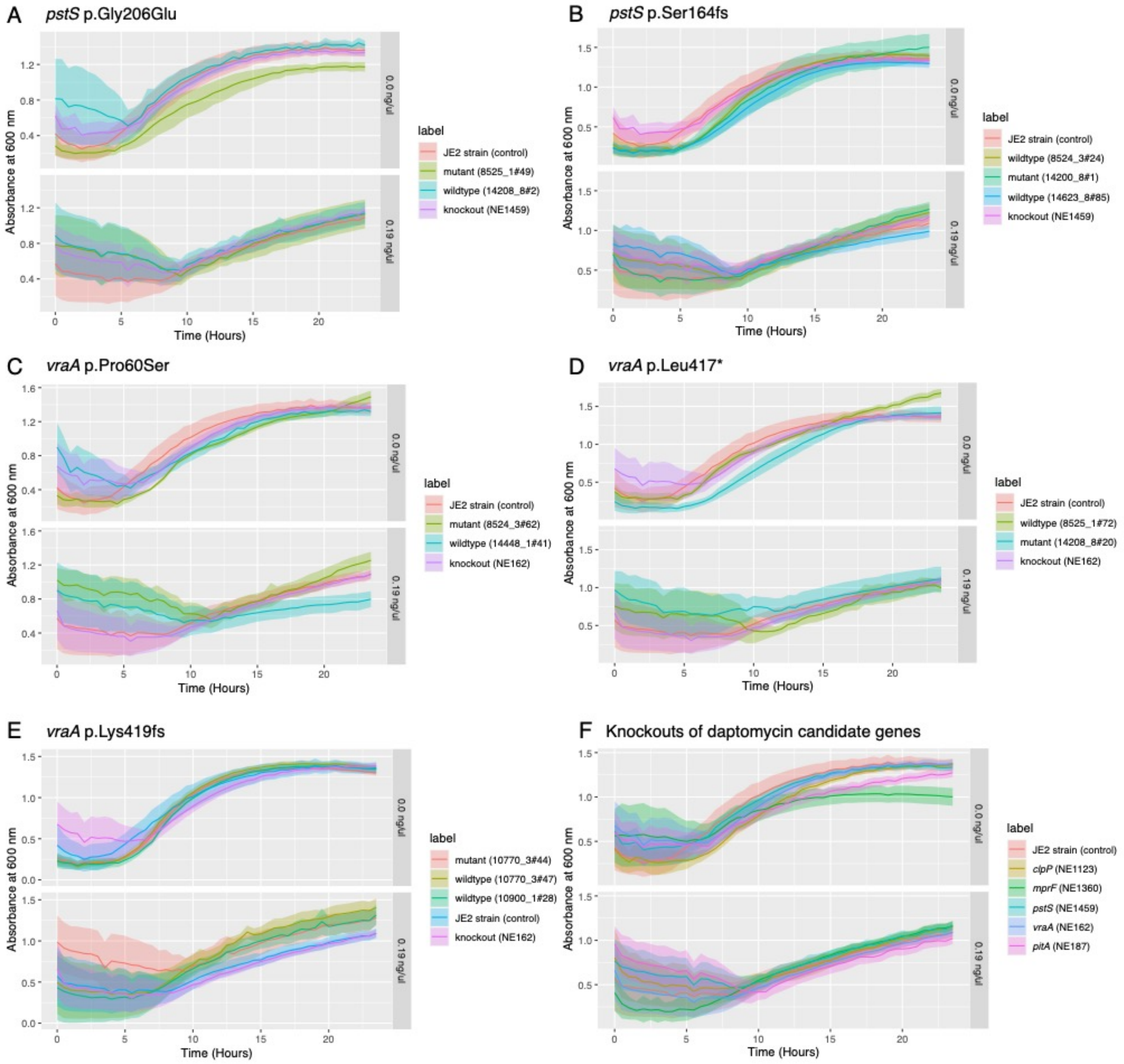

Panels A to E. Growth curves of *S. aureus* isolates carrying natural mutations in *pstS* and *vraA* genes in the absence and presence of sub-inhibitory concentration of daptomycin (0.19 µg/mL). As controls we grew wildtype *S. aureus* isolates from the same host lacking the investigated mutation, a transposon knockout of the investigated gene (*pstS* or *vraA*), and the *S. aureus* strain (JE2) used to build the transposon library. Panel F. Growth curves of *S. aureus* transposon knockouts in daptomycin-candidate genes. The values of absorbance at 600 nm are plotted in the y-axis as a function of time (in hours). Daptomycin concentration is indicated at the right edge of each plot.

Supplementary Figure 8. Loci enriched for protein-altering mutations in colonising isolates of the extended dataset

A CDS

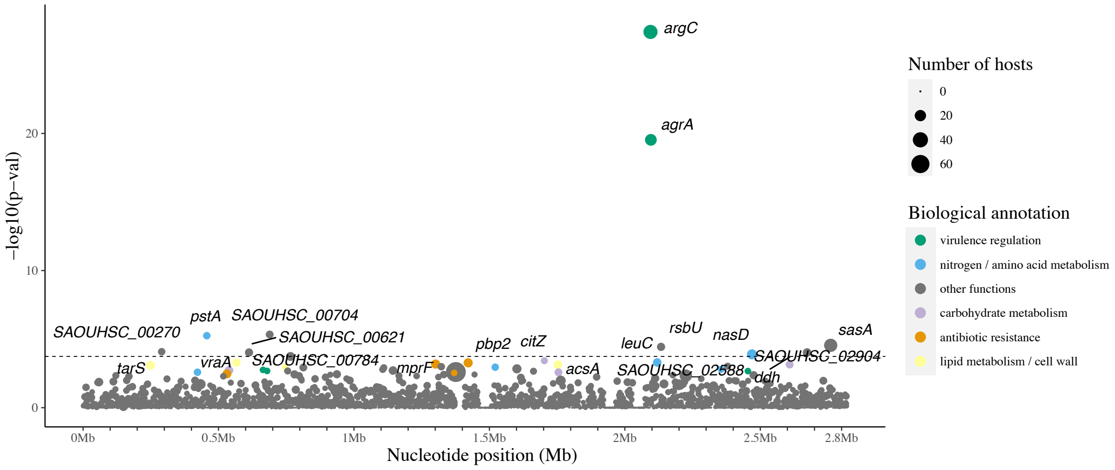

B Transcription units (operons)

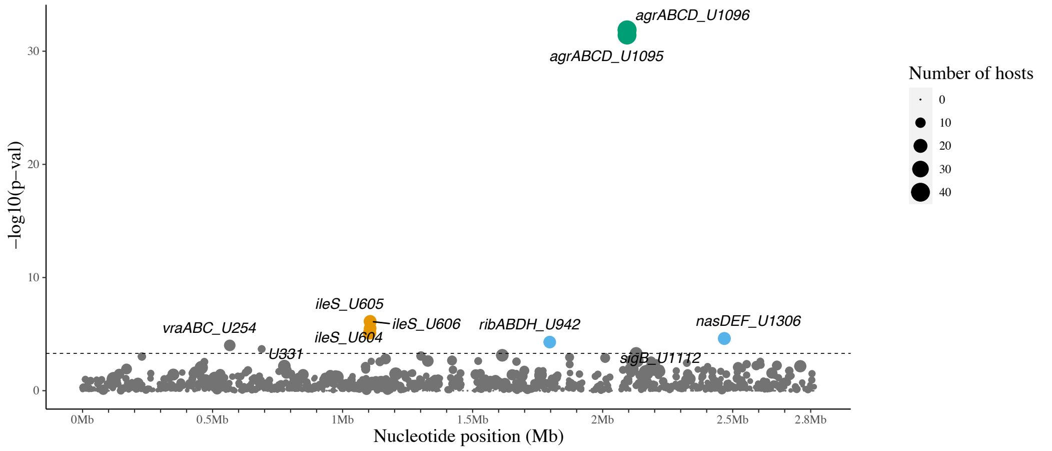

C Metabolic sub-modules

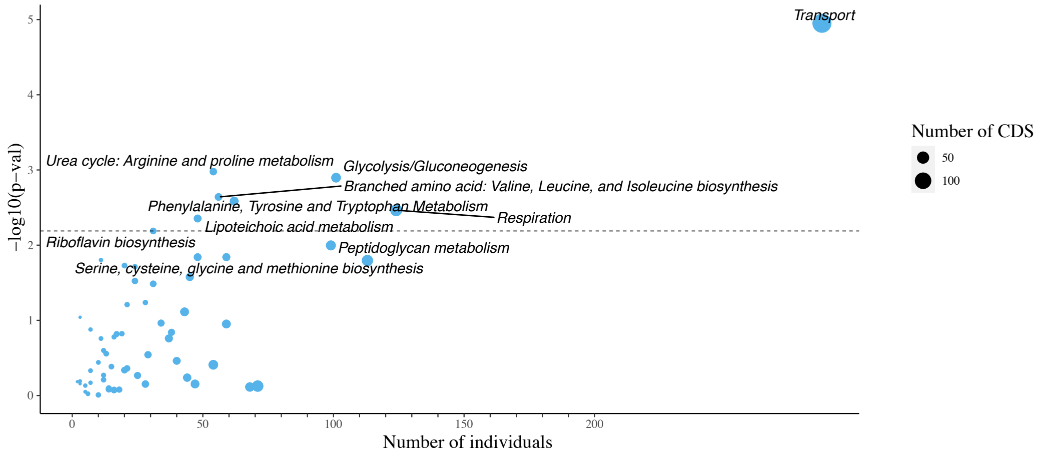

See footnote of Figure 2 for legend information.

Supplementary Figure 9. Positions of protein altering mutations on SraP.

A

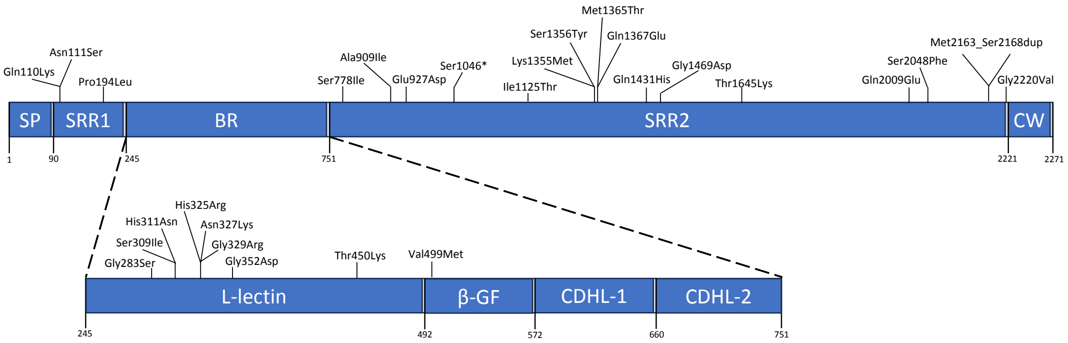

B

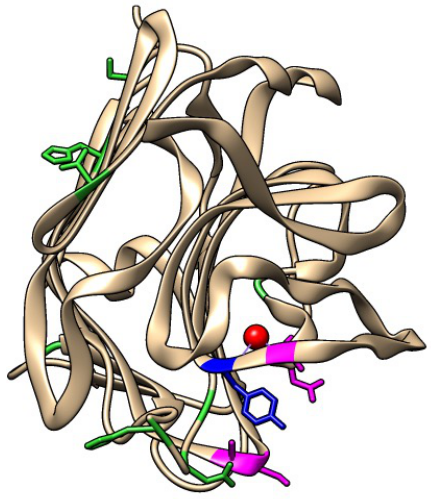

A) The domain organisation of SraP indicating the location of amino-acid changes arising from missense mutations. B) The structure of the SraP L-lectin domain showing residues comprising the predicted N-acetylneuraminic acid binding site and the amino-acid substitutions arising from missense mutations. Y367 (blue) is critical for binding to N-acetylneuraminic acid, while modelling performed by Yang *et al.*, 2014[62] suggests that the residues shown in magenta (S293, G477, A478, N369, and N347) contribute to the sugar-binding site. The bound Ca^{2+} ion is shown in red. The 8 amino-acids altered by missense mutations within the L-lectin domain are shown in lime green. This image was generated from the structure of SraP L-lectin domain (Protein Data Bank accession code 4M01, Chain A) using UCSF Chimera version 1.16.

Supplementary References

1. Coll, F. *et al.* Longitudinal genomic surveillance of MRSA in the UK reveals transmission patterns in hospitals and the community. *Science Translational Medicine* **9**, eaak9745 (2017).

2. Price, J. R. *et al.* Transmission of *Staphylococcus aureus* between health-care workers, the environment, and patients in an intensive care unit: a longitudinal cohort study based on whole-genome sequencing. *The Lancet Infectious Diseases* **17**, 207–214 (2017).

3. Young, B. C. *et al.* Severe infections emerge from commensal bacteria by adaptive evolution. *eLife* **6**, 1–25 (2017).

4. Chow, A. *et al.* MRSA Transmission Dynamics Among Interconnected Acute, Intermediate-Term, and Long-Term Healthcare Facilities in Singapore. *Clinical Infectious Diseases* **64**, S76–S81 (2017).

5. Price, J. R. *et al.* Whole-Genome Sequencing Shows That Patient-to-Patient Transmission Rarely Accounts for Acquisition of *Staphylococcus aureus* in an Intensive Care Unit. *Clinical Infectious Diseases* **58**, 609–618 (2014).

6. Tong, S. Y. C. *et al.* Genome sequencing defines phylogeny and spread of methicillin-resistant *Staphylococcus aureus* in a high transmission setting. *Genome Res* **25**, 111–118 (2015).

7. Harkins, C. P. *et al.* The Microevolution and Epidemiology of *Staphylococcus aureus* Colonization during Atopic Eczema Disease Flare. *Journal of Investigative Dermatology* **138**, 336–343 (2018).

8. Tosas Auguet, O. *et al.* Evidence for Community Transmission of Community-Associated but Not Health-Care-Associated Methicillin-Resistant *Staphylococcus aureus* Strains Linked to Social and Material Deprivation:

Spatial Analysis of Cross-sectional Data. *PLOS Medicine* **13**, e1001944 (2016).

9. Harrison, E. M. *et al.* Transmission of methicillin-resistant *Staphylococcus aureus* in long-term care facilities and their related healthcare networks. *Genome Medicine* **8**, 102 (2016).

10. Paterson, G. K. *et al.* Capturing the cloud of diversity reveals complexity and heterogeneity of MRSA carriage, infection and transmission. *Nature Communications* **6**, 6560 (2015).

11. Kumar, N. *et al.* Evaluation of a fully automated bioinformatics tool to predict antibiotic resistance from MRSA genomes. *Journal of Antimicrobial Chemotherapy* **75**, 1117–1122 (2020).

12. Murthy, M. H., Olson, M. E., Wickert, R. W., Fey, P. D. & Jalali, Z. Daptomycin non-susceptible meticillin-resistant *Staphylococcus aureus* USA 300 isolate. *Journal of Medical Microbiology* **57**, 1036–1038 (2008).

13. Friedman, L., Alder, J. D. & Silverman, J. A. Genetic Changes That Correlate with Reduced Susceptibility to Daptomycin in *Staphylococcus aureus*. *Antimicrobial Agents and Chemotherapy* **50**, 2137–2145 (2006).

14. Thitiananpakorn, K. *et al.* Association of mprF mutations with cross-resistance to daptomycin and vancomycin in methicillin-resistant *Staphylococcus aureus* (MRSA). *Scientific Reports* **10**, 1–15 (2020).

15. Mishra, N. N., Rubio, A., Nast, C. C. & Bayer, A. S. Differential Adaptations of Methicillin-Resistant *Staphylococcus aureus* to Serial In Vitro Passage in Daptomycin: Evolution of Daptomycin Resistance and Role of Membrane Carotenoid Content and Fluidity. *International Journal of Microbiology* **2012**, 1–6 (2012).

16. Capone, A. *et al.* In vivo development of daptomycin resistance in vancomycin-susceptible methicillin-resistant *Staphylococcus aureus* severe infections previously treated with glycopeptides. *European Journal of Clinical Microbiology & Infectious Diseases* **35**, 625–631 (2016).

17. Cameron, D. R. *et al.* Impact of daptomycin resistance on *Staphylococcus aureus* virulence. *Virulence* **6**, 127–131 (2015).

18. Sulaiman, J. E. & Lam, H. Novel Daptomycin Tolerance and Resistance Mutations in Methicillin-Resistant *Staphylococcus aureus* from Adaptive Laboratory Evolution. *mSphere* **6**, (2021).

19. Berti, A. D. *et al.* Heterogeneity of Genetic Pathways toward Daptomycin Nonsusceptibility in *Staphylococcus aureus* Determined by Adjunctive Antibiotics. *Antimicrobial Agents and Chemotherapy* **59**, 2799–2806 (2015).

20. Nakamura, M. *et al.* Single nucleotide polymorphism leads to daptomycin resistance causing amino acid substitution—T345I in MprF of clinically isolated MRSA strains. *PLOS ONE* **16**, e0245732 (2021).

21. Lin, Y.-T. *et al.* Emergence of a small colony variant of vancomycin-intermediate *Staphylococcus aureus* in a patient with septic arthritis during long-term treatment with daptomycin. *Journal of Antimicrobial Chemotherapy* **71**, 1807–1814 (2016).

22. Peleg, A. Y. *et al.* Whole Genome Characterization of the Mechanisms of Daptomycin Resistance in Clinical and Laboratory Derived Isolates of *Staphylococcus aureus*. *PLoS ONE* **7**, e28316 (2012).

23. Yamaguchi, T. *et al.* Evolution and Single-Nucleotide Polymorphisms in Methicillin-Resistant *Staphylococcus aureus* Strains with Reduced Susceptibility to Vancomycin and Daptomycin, Based on Determination of the

Complete Genome. *Antimicrobial Agents and Chemotherapy* **59**, 3585–3587 (2015).

24. Yang, S. J. *et al.* Regulation of mprF in daptomycin-nonsusceptible *Staphylococcus aureus* strains. *Antimicrobial Agents and Chemotherapy* **53**, 2636–2637 (2009).
25. Bæk, K. T. *et al.* Stepwise Decrease in Daptomycin Susceptibility in Clinical *Staphylococcus aureus* Isolates Associated with an Initial Mutation in rpoB and a Compensatory Inactivation of the clpX Gene. *Antimicrobial Agents and Chemotherapy* **59**, 6983–6991 (2015).
26. Patel, D. *et al.* Mechanisms of in-vitro-selected daptomycin-non-susceptibility in *Staphylococcus aureus*. *International Journal of Antimicrobial Agents* **38**, 442–446 (2011).
27. Jiang, S. *et al.* The role of mprF mutations in “see-saw effect” of Daptomycin-resistant methicillin-resistant *Staphylococcus aureus* isolates. *Antimicrobial Agents and Chemotherapy* (2021) doi:10.1128/AAC.01295-21.
28. Hao, S. *et al.* Genomic Profiling of Evolving Daptomycin Resistance in a Patient with Recurrent Staphylococcus argenteus Sepsis. *Antimicrobial Agents and Chemotherapy* **64**, (2020).
29. Weber, R. E. *et al.* Genome-Wide Association Studies for the Detection of Genetic Variants Associated With Daptomycin and Ceftaroline Resistance in *Staphylococcus aureus*. *Frontiers in Microbiology* **12**, 1–15 (2021).
30. Mehta, S. *et al.* VraSR Two-Component Regulatory System Contributes to mprF -Mediated Decreased Susceptibility to Daptomycin in In Vivo -Selected Clinical Strains of Methicillin-Resistant *Staphylococcus aureus*. *Antimicrobial Agents and Chemotherapy* **56**, 92–102 (2012).

31. Hines, K. M. *et al.* Characterization of the Mechanisms of Daptomycin Resistance among Gram-Positive Bacterial Pathogens by Multidimensional Lipidomics. *mSphere* **2**, (2017).

32. Sabat, A. J. *et al.* Daptomycin Resistant *Staphylococcus aureus* Clinical Strain With Novel Non-synonymous Mutations in the mprF and vraS Genes: A New Insight Into Daptomycin Resistance. *Frontiers in Microbiology* **9**, (2018).

33. Okado, J. B., Avaca-Crusca, J. S., Oliveira, A. L., Dabul, A. N. G. & Camargo, I. L. B. da C. Daptomycin and vancomycin heteroresistance revealed among CC5-SCCmecII MRSA clone and in vitro evaluation of treatment alternatives. *Journal of Global Antimicrobial Resistance* **14**, 209–216 (2018).

34. Vidaillac, C. *et al.* Alternative Mutational Pathways to Intermediate Resistance to Vancomycin in Methicillin-Resistant *Staphylococcus aureus*. *The Journal of Infectious Diseases* **208**, 67–74 (2013).

35. Iwata, Y. *et al.* Down-regulation of the two-component system and cell-wall biosynthesis-related genes was associated with the reversion to daptomycin susceptibility in daptomycin non-susceptible methicillin-resistant *Staphylococcus aureus*. *European Journal of Clinical Microbiology & Infectious Diseases* **36**, 1839–1845 (2017).

36. Guérillot, R. *et al.* Convergent Evolution Driven by Rifampin Exacerbates the Global Burden of Drug-Resistant *Staphylococcus aureus*. *mSphere* **3**, (2018).

37. Cui, L. *et al.* An RpoB Mutation Confers Dual Heteroresistance to Daptomycin and Vancomycin in *Staphylococcus aureus*. *Antimicrobial Agents and Chemotherapy* **54**, 5222–5233 (2010).

38. Basco, M. D. S. *et al.* Reduced vancomycin susceptibility and increased macrophage survival in *Staphylococcus aureus* strains sequentially isolated

from a bacteraemic patient during a short course of antibiotic therapy. *Journal of Medical Microbiology* **68**, 848–859 (2019).

39. Taglialegna, A., Varela, M. C., Rosato, R. R. & Rosato, A. E. VraSR and Virulence Trait Modulation during Daptomycin Resistance in Methicillin-Resistant *Staphylococcus aureus* Infection. *mSphere* **4**, (2019).

40. Su, J. *et al.* A novel mutation in the vraS gene of *Staphylococcus aureus* contributes to reduce susceptibility against daptomycin. *The Journal of Antibiotics* **68**, 646–648 (2015).

41. Berscheid, A. *et al.* Generation of a vancomycin-intermediate *Staphylococcus aureus* (VISA) strain by two amino acid exchanges in VraS. *Journal of Antimicrobial Chemotherapy* **69**, 3190–3198 (2014).

42. Miller, C. R., Monk, J. M., Szubin, R. & Berti, A. D. Rapid resistance development to three antistaphylococcal therapies in antibiotic-tolerant *Staphylococcus aureus* bacteremia. *PLoS ONE* **16**, 1–15 (2021).

43. Jiang, J.-H. *et al.* Antibiotic resistance and host immune evasion in *Staphylococcus aureus* mediated by a metabolic adaptation. *Proceedings of the National Academy of Sciences* **116**, 3722–3727 (2019).

44. Cafiso, V. *et al.* dltA overexpression: A strain-independent keystone of daptomycin resistance in methicillin-resistant *Staphylococcus aureus*. *International Journal of Antimicrobial Agents* **43**, 26–31 (2014).

45. Yang, B., Yao, H., Li, D. & Liu, Z. The phosphatidylglycerol phosphate synthase PgsA utilizes a trifurcated amphipathic cavity for catalysis at the membrane-cytosol interface. *Current Research in Structural Biology* **3**, 312–323 (2021).

46. Kang, K.-M. *et al.* Phenotypic and genotypic correlates of daptomycin-resistant

methicillin-susceptible *Staphylococcus aureus* clinical isolates. *Journal of Microbiology* **55**, 153–159 (2017).

47. Hall, M. D. *et al.* Improved characterisation of MRSA transmission using within-host bacterial sequence diversity. *eLife* **8**, 124–131 (2019).

48. Chow, A. *et al.* Comparative epidemiology and factors associated with major healthcare-associated methicillin-resistant *Staphylococcus aureus* clones among interconnected acute-, intermediate- and long-term healthcare facilities in Singapore. *Clinical Microbiology and Infection* **27**, 785.e9-785.e16 (2021).

49. van Tonder, A. J. *et al.* Colonization and transmission of *Staphylococcus aureus* in schools: a citizen science project. *Microbial Genomics* **9**, (2023).

50. Nakamura, Y. *et al.* Staphylococcus Agr virulence is critical for epidermal colonization and associates with atopic dermatitis development. *Science Translational Medicine* **12**, (2020).

51. Effelsberg, N. *et al.* Genotypic Characterization of Livestock-Associated Methicillin-Resistant *Staphylococcus aureus* Isolates of Clonal Complex 398 in Pigsty Visitors: Transient Carriage or Persistence? *Journal of Clinical Microbiology* **58**, (2019).

52. Holt, D. C. *et al.* Longitudinal whole-genome based comparison of carriage and infection associated *Staphylococcus aureus* in northern Australian dialysis clinics. *PLOS ONE* **16**, e0245790 (2021).

53. Ingham, A. C. *et al.* Dynamics of the Human Nasal Microbiota and *Staphylococcus aureus* CC398 Carriage in Pig Truck Drivers across One Workweek. *Applied and Environmental Microbiology* **87**, (2021).

54. Lydecker, A. D. *et al.* Targeted gown and glove use to prevent *Staphylococcus aureus* acquisition in community-based nursing homes: A pilot study. *Infection*

Control & Hospital Epidemiology **42**, 448–454 (2021).

55. Lee, R. S. *et al.* 561. Genomic Epidemiology of Methicillin-Resistant *Staphylococcus aureus* in Two Cohorts of High-Risk Military Trainees. *Open Forum Infectious Diseases* **6**, S266–S266 (2019).
56. Popovich, K. J. *et al.* MRSA Transmission in Intensive Care Units: Genomic Analysis of Patients, Their Environments, and Healthcare Workers. *Clinical Infectious Diseases* **72**, 1879–1887 (2021).
57. Popovich, K. J. *et al.* Frequent Methicillin-Resistant *Staphylococcus aureus* Introductions Into an Inner-city Jail: Indications of Community Transmission Networks. *Clinical Infectious Diseases* **71**, 323–331 (2020).
58. Sansom, S. E. *et al.* Genomic Update of Phenotypic Prediction Rule for Methicillin-Resistant *Staphylococcus aureus* (MRSA) USA300 Discloses Jail Transmission Networks with Increased Resistance. *Microbiology Spectrum* **9**, (2021).
59. Key, F. M. *et al.* On-person adaptive evolution of *Staphylococcus aureus* during treatment for atopic dermatitis. *Cell Host & Microbe* **31**, 593-603.e7 (2023).
60. Annavajhala, M. K. *et al.* Genomic and Epidemiological Features of Two Dominant Methicillin-Susceptible *Staphylococcus aureus* Clones from a Neonatal Intensive Care Unit Surveillance Effort. *mSphere* **7**, (2022).
61. Raghuram, V. *et al.* Comparison of genomic diversity between single and pooled *Staphylococcus aureus* colonies isolated from human colonization cultures. *Microbial Genomics* **9**, (2023).
62. Yang, Y.-H. *et al.* Structural Insights into SraP-Mediated *Staphylococcus aureus* Adhesion to Host Cells. *PLoS Pathogens* **10**, e1004169 (2014).
