# Supplementary material for: The mutational landscape of Staphylococcus aureus during colonisation
Source: Nat Commun. 2025 Jan 13;16:302. doi: 10.1038/s41467-024-55186-x (PMC11730646; doi:10.1038/s41467-024-55186-x)
Supplement: Supplementary file 2 — Description of Additional Supplementary Files [file 41467_2024_55186_MOESM2_ESM.pdf]

Description of Additional Supplementary Files

Supplementary Data 1. Isolate accession and metadata

Supplementary Data 2. Hits of mutation enrichment analyses

Supplementary Data 3. Raw growth curves measurements and growth parameters obtained under different nitrogen sources

Supplementary Data 4. Raw growth curves measurements and growth parameters obtained with and without daptomycin

Supplementary Data 5. Mutations detected by Breseq between the pairs of related isolates from the same host tested *in vitro*
