# Supplementary material for: The mutational landscape of Staphylococcus aureus during colonisation
Source: Nat Commun. 2025 Jan 13;16:302. doi: 10.1038/s41467-024-55186-x (PMC11730646; doi:10.1038/s41467-024-55186-x)
Supplement: Supplementary file 8 — Reporting Summary [file 41467_2024_55186_MOESM8_ESM.pdf]

nature portfolio

Corresponding author(s): Francesc Coll & Ewan M Harrison

Last updated by author(s): Oct 18, 2024

# Reporting Summary

Nature Portfolio wishes to improve the reproducibility of the work that we publish. This form provides structure for consistency and transparency in reporting. For further information on Nature Portfolio policies, see our Editorial Policies and the Editorial Policy Checklist.

## Statistics

For all statistical analyses, confirm that the following items are present in the figure legend, table legend, main text, or Methods section.

| n/a | Confirmed | |
| --- | --- | --- |
| ☐ | ☒ | The exact sample size (*n*) for each experimental group/condition, given as a discrete number and unit of measurement |
| ☐ | ☒ | A statement on whether measurements were taken from distinct samples or whether the same sample was measured repeatedly |
| ☐ | ☒ | The statistical test(s) used AND whether they are one- or two-sided<br>*Only common tests should be described solely by name; describe more complex techniques in the Methods section.* |
| ☐ | ☒ | A description of all covariates tested |
| ☐ | ☒ | A description of any assumptions or corrections, such as tests of normality and adjustment for multiple comparisons |
| ☐ | ☒ | A full description of the statistical parameters including central tendency (e.g. means) or other basic estimates (e.g. regression coefficient) AND variation (e.g. standard deviation) or associated estimates of uncertainty (e.g. confidence intervals) |
| ☐ | ☒ | For null hypothesis testing, the test statistic (e.g. *F*, *t*, *r*) with confidence intervals, effect sizes, degrees of freedom and *P* value noted<br>*Give P values as exact values whenever suitable.* |
| ☒ | ☐ | For Bayesian analysis, information on the choice of priors and Markov chain Monte Carlo settings |
| ☒ | ☐ | For hierarchical and complex designs, identification of the appropriate level for tests and full reporting of outcomes |
| ☐ | ☒ | Estimates of effect sizes (e.g. Cohen's *d*, Pearson's *r*), indicating how they were calculated |

*Our web collection on statistics for biologists contains articles on many of the points above.*

## Software and code

Policy information about availability of computer code

| | |
| --- | --- |
| Data collection | NCBI's Entrez Direct unix command line tools version 13.9 was used to extract individual run accessions and metadata from BioProject accessions |
| Data analysis | The following open-source bioinformatics tools were used:<br>fastqcheck v1.1 (https://github.com/VertebrateResequencing/fastqcheck)<br>Spades v.3.12.0<br>QUAST v5.0.1<br>Bowtie2 v1.2.2<br>Kraken v2.1.2<br>AMRFinderPlus93 v3.11.11 (AMRFinder database v2023-08-08.2)<br>SMALT v0.7.6 (http://www.sanger.ac.uk/resources/software/smalt/)<br>bcftools v0.1.19<br>pairsnp v0.0.1<br>Roary v3.11.1<br>RAxML v8.2.8<br>Snippy v4.3.3<br>PastML v1.9.20<br>bcftools v1.9<br>Blastn v2.8.1+<br>dustmasker v1.0.0<br>SnpEff v4.3 |

Breseq v0.39.0
R version 3.4.1

For manuscripts utilizing custom algorithms or software that are central to the research but not yet described in published literature, software must be made available to editors and reviewers. We strongly encourage code deposition in a community repository (e.g. GitHub). See the Nature Portfolio guidelines for submitting code & software for further information.

## Data

Policy information about availability of data

All manuscripts must include a data availability statement. This statement should provide the following information, where applicable:

- Accession codes, unique identifiers, or web links for publicly available datasets
- A description of any restrictions on data availability
- For clinical datasets or third party data, please ensure that the statement adheres to our policy

We identified available collections of S. aureus genomes with multiple carriage isolates sequenced from the same human individual. The NCBI Short Read Archive (SRA) was systematically queried on June 2019 to identify BioProjects that met the following criteria: contained S. aureus genomic sequences, could be linked to a publication, included genomes of clinical isolates, clinical sources were known, multiple colonising isolates per host were sequenced, and host IDs were available. BioProjects with less than 70 S. aureus isolate genomes were discarded. BioProjects including genomic data other than S. aureus clinical isolate genomes; that is, animal strains, mutagenesis experiments, RNAseq or microbiomes were discarded. BioProjects with multiple isolates per host but without host identifiers could not be included. Only isolates from colonisation specimens were kept, that is, from multi-site screens and typical colonising anatomical sites (nose, armpit, groin, perineum and throat). The whole genome sequences of the isolate collections used in this study are available on European Nucleotide Archive (ENA) under the accessions listed in Supplementary Data 1, which also includes isolate metadata. All scripts necessary to run the described analyses are available on GitHub (https://github.com/francesccoll/staph-adaptive-mutations). The full list of protein-coding regions, transcriptional units and metabolic processes enriched by protein-altering mutations can be found in Supplementary Data 2. Supplementary Data 3 and 4 include the data of bacterial growth curves.

## Research involving human participants, their data, or biological material

Policy information about studies with human participants or human data. See also policy information about sex, gender (identity/presentation), and sexual orientation and race, ethnicity and racism.

| | |
|---|---|
| Reporting on sex and gender | No human participants were recruited as part of this study. Only the whole-genome sequences of S. aureus isolates obtained from published studies, in addition to site and date of isolation of these, were analyzed in this study. |
| Reporting on race, ethnicity, or other socially relevant groupings | Not applicable. |
| Population characteristics | Not applicable. |
| Recruitment | Not applicable. |
| Ethics oversight | Not applicable. |

Note that full information on the approval of the study protocol must also be provided in the manuscript.

# Field-specific reporting

Please select the one below that is the best fit for your research. If you are not sure, read the appropriate sections before making your selection.

☐ Life sciences ☐ Behavioural & social sciences ☒ Ecological, evolutionary & environmental sciences

For a reference copy of the document with all sections, see nature.com/documents/nr-reporting-summary-flat.pdf

# Ecological, evolutionary & environmental sciences study design

All studies must disclose on these points even when the disclosure is negative.

| | |
|---|---|
| Study description | This study applied a convergent evolution approach to identify putative adaptive genetic variation in S. aureus strains acquired during episodes of colonisation. |
| Research sample | Existing datasets of Staphylococcus aureus colonisation isolates. Published studies that sequenced multiple S. aureus isolate genomes from colonisation specimens were kept, that is, from multi-site screens and typical colonising anatomical sites (nose, armpit, groin, perineum and throat). |
| Sampling strategy | Not applicable. See comments below. |
| Data collection | The dataset compiled in this study is made up of multiple colonization S. aureus isolates sequenced per host extracted from a total of 25 published studies. Most studies collected S. aureus isolates in the context of genomic surveillance studies, most often in health- |

care settings. See Supplementary Data 1 for isolate and study information.

| | |
|---|---|
| Timing and spatial scale | Published studies report specimen collection dates ranging from 2008 to 2018 (See Supplementary Data 1). In terms of timing between samples, 556 individuals (70.3%) had their isolates collected within two months, 126 (15.9%) between two to six months, and 95 (12.0%) between six to twelve months, and 14 (1.8%) more than a year apart. |
| Data exclusions | BioProjects with less than 70 S. aureus isolate genomes were discarded. BioProjects including genomic data other than S. aureus clinical isolate genomes; that is, animal strains, mutagenesis experiments, RNAseq or microbiomes were discarded. BioProjects with multiple isolates per host but without host identifiers could not be included. Only isolates from colonisation specimens were kept, that is, from multi-site screens and typical colonizing anatomical sites (nose, armpit, groin, perineum and throat). Low-quality genomes were excluded from further analysis applying the following thresholds: S. aureus reads < 80%, N50 < 10000, contigs smaller than 1 kb contributing to >15% of the total assembly length, total assembly length outside of the median ± one standard deviation, or >1500 polymorphic sites. |
| Reproducibility | In terms of genomic analyses, to strengthen our initial findings (those obtained when analyzing the initial dataset of 3,060 isolates from 791 individuals), we increased sample size of the dataset by an additional 4,090 isolate genomes from 731 individuals and 15 different studies. We applied the same curation, genomic and QC methodological steps to keep only high-quality and clonal genomes of the same individual from colonisation sources. Growth curves were conducted with three biological and three technical replicates (9 replicated in total). |
| Randomization | Not applicable. |
| Blinding | Not applicable. |

Did the study involve field work? ☐ Yes ☒ No

# Reporting for specific materials, systems and methods

We require information from authors about some types of materials, experimental systems and methods used in many studies. Here, indicate whether each material, system or method listed is relevant to your study. If you are not sure if a list item applies to your research, read the appropriate section before selecting a response.

## Materials & experimental systems

| n/a | Involved in the study | |
|---|---|---|
| ☒ | ☐ | Antibodies |
| ☒ | ☐ | Eukaryotic cell lines |
| ☒ | ☐ | Palaeontology and archaeology |
| ☒ | ☐ | Animals and other organisms |
| ☒ | ☐ | Clinical data |
| ☒ | ☐ | Dual use research of concern |
| ☒ | ☐ | Plants |

## Methods

| n/a | Involved in the study | |
|---|---|---|
| ☒ | ☐ | ChIP-seq |
| ☒ | ☐ | Flow cytometry |
| ☒ | ☐ | MRI-based neuroimaging |

# Plants

| | |
|---|---|
| Seed stocks | Not applicable |
| Novel plant genotypes | Not applicable |
| Authentication | Not applicable |
